# Decreased integration of default-mode network during a working memory task in schizophrenia with severe attention deficits

**DOI:** 10.3389/fncel.2022.1006797

**Published:** 2022-11-08

**Authors:** Peng Cheng, Zhening Liu, Jun Yang, Fuping Sun, Zebin Fan, Jie Yang

**Affiliations:** ^1^Department of Psychiatry, The Second Xiangya Hospital of Central South University, Changsha, China; ^2^National Clinical Research Center for Mental Disorders, Changsha, China

**Keywords:** working memory, attention deficits, brain networks, graph theory, N-back task

## Abstract

**Background:**

Working memory (WM) and attention deficits are both important features of schizophrenia. WM is closely related to attention, for it acted as an important characteristic in activating and manipulating WM. However, the knowledge of neural mechanisms underlying the relationship between WM and attention deficits in schizophrenia is poorly investigated.

**Methods:**

Graph theory was used to examine the network topology at the whole-brain and large-scale network levels among 125 schizophrenia patients with different severity of attention deficits (65 mild attention deficits; 46 moderate attention deficits; and 14 severe attention deficits) and 53 healthy controls (HCs) during an N-back WM task. These analyses were repeated in the same participants during the resting state.

**Results:**

In the WM task, there were omnibus differences in small-worldness and normalized clustering coefficient at a whole-brain level and normalized characterized path length of the default-mode network (DMN) among all groups. *Post hoc* analysis further indicated that all patient groups showed increased small-worldness and normalized clustering coefficient of the whole brain compared with HCs, and schizophrenia with severe attention deficits showed increased normalized characterized path length of the DMN compared with schizophrenia with mild attention deficits and HCs. However, these observations were not persisted under the resting state. Further correlation analyses indicated that the increased normalized characterized path length of the DMN was correlated with more severe attentional deficits and poorer accuracy of the WM task.

**Conclusion:**

Our research demonstrated that, compared with the schizophrenia patients with less attention deficits, disrupted integration of the DMN may more particularly underlie the WM deficits in schizophrenia patients with severe attention deficits.

## Introduction

Working memory (WM) is a complex cognitive process involved in encoding, storing, and retrieving information steps (Baddeley, 1986). It is closely related to attention, which is a crucial cognitive ability that enables humans to select goal-relevant information from external sensory stimuli in the environment. Previous studies have reported that impairments in attentional selection can have detrimental effects on WM encoding, specifically when top-down processes are involved (Baddeley, 1996). Meanwhile, the ability to resist interference is also crucial for maintaining the stability of memory representation over time and extracting information from WM (Berti and Schröger, 2003; Lorenc et al., 2021). Thus, attention deficits are likely to affect WM at different processing.

Both attention and WM deficits are common neurocognitive impairments of schizophrenia. They are persisted in schizophrenia even after systematic treatment, which would contribute to frequent relapses (Hui et al., 2016) and companied prolonged illness burden (Park et al., 1999). However, current treatments are mainly aimed at addressing the positive symptoms, with minor effects on neurocognitive deficits. Therefore, understanding the neural mechanism of attention and WM impairments is vitally important for developing new treatments that can impact the long-term functional outcome of schizophrenia.

Extensive previous studies have investigated the neural mechanism of WM dysfunction in patients with schizophrenia and revealed that the hyperactivity (i.e., failure to deactivate) within the default-mode network (DMN) (Pomarol-Clotet et al., 2008; Whitfield-Gabrieli et al., 2009) was companied with hypoactivity of the task-positive network (TPN) (Meyer-Lindenberg et al., 2001) during the WM task. Reduced suppression of the DMN in schizophrenia can be interpreted as a failure to allocate attentional resources to the current task, with consequent impairment in task performance (Whitfield-Gabrieli and Ford, 2012). The anticorrelation could be regarded as an alternating balance of attention to internal thoughts (associated with DMN) and external feelings (associated with TPN). This greater anticorrelation, associated with superior cognitive task performance, seemed to be reduced in schizophrenia patients (Kelly et al., 2008; Hampson et al., 2010; Liu et al., 2012). Especially, Whitfield-Gabrieli et al. (2009) have demonstrated that compared with healthy controls (HCs), patients with schizophrenia showed reduced anticorrelation between the medial prefrontal cortex (MPFC), a crucial component of DMN, and the dorsolateral prefrontal cortex (DLPFC), a well-known important region for TPN.

Previous studies have also explored the neural mechanisms of attention deficits in patients with schizophrenia. It was reported a significant uncoupling between attention performance and mean regional homogeneity in the left middle frontal gyrus, right superior/inferior parietal lobe (IPL), and right angular gyrus (AG) in patients with schizophrenia compared with HCs (Hong et al., 2019). The decreased integrity of the bilateral cingulum and splenium may underlie the impaired attention in schizophrenia (Abdolalizadeh et al., 2020).

Although these studies have investigated the neural mechanisms of the WM and attention deficits in schizophrenia and obtained some progress these years, however, the potential mechanism of attention deficits affecting WM performance in schizophrenia is still unclear. The WM can be defined as a certain subset of the process encompassed by the attention, which is composed of multiple neuropsychological and/or clinical processes (Fan et al., 2003; Pessoa et al., 2003), and the attention deficits in schizophrenia can have effects on their WM performance (Dickinson et al., 2007; Luck and Gold, 2008). It is meaningful to investigate the possible connection between WM and attention deficits among schizophrenias.

Mathematical graph theory employed in neuroimaging data can represent the topological structure of the brain connectome and infer its neural efficiency (Bullmore and Sporns, 2009; Rubinov and Sporns, 2010; Fornito et al., 2011). Compared with the traditional functional connectivity analysis that mainly investigates the synchrony between different brain regions (Pomarol-Clotet et al., 2008; Whitfield-Gabrieli et al., 2009), the graph theory could further indicate the efficiency of information processing at either the subnetwork level or the whole-brain level (van den Heuvel and Fornito, 2014). Moreover, previous related studies have suggested that human brain networks are organized in an efficient small-world manner (i.e., a highly clustered/segregated neighborhood of brain regions, with occasional integrative long-distance connections) (Tononi et al., 1994; Bullmore and Sporns, 2009). Some studies have explored the brain connectome topology in patients with schizophrenia during resting state and reported less efficient organization in patients compared with HCs (Fornito et al., 2012; Hadley et al., 2016; Yu et al., 2017; Jiang et al., 2022). Some studies go further to investigate the brain connectome topology of schizophrenias during tasks. For example, our previous work (Yang et al., 2020) demonstrated a putative mechanistic link between whole-brain connectome topology and impaired performance in schizophrenia during the WM task. It suggested that the task-dependent increased small-worldness relates to, but remains inefficient in, improving the performance above par in patients with severe negative symptoms. However, to our knowledge, no studies have investigated the brain connectome topology in schizophrenia with attention deficits under the WM task.

This study aimed to use the graph theory method to investigate the topology properties of the functional connectome at different granularity (i.e., whole brain and subnetworks) of schizophrenia patients with different attention deficit severity during the WM task. Based on the results of our prior work (Yang et al., 2020), we speculated that all patients with schizophrenia, despite of their different attentional deficit severity, would show increased small-worldness of the whole-brain functional connectome, and this increment is induced by the elevation of the clustering coefficient. Meanwhile, based on the abnormal activation of DMN and TPN among schizophrenias during the WM task claimed by previous studies (Moraschi et al., 2020; Yuan et al., 2021), we also speculated that the topology property of subnetworks in patients with schizophrenia might also change when processing the WM task. In addition, based on the close association between WM and attention deficits, we further hypothesized that schizophrenia patients with more severe attention deficits were more likely to their special topology alterations of their brain connectome than other patients.

## Materials and methods

### Participants

Participants in the current study were enrolled in the Second Xiangya Hospital, Central South University, during 2009–2014. A total of 142 patients with schizophrenia and 59 HCs were enrolled. All the participants were right-handed native Chinese speakers. Written informed consent was obtained from participants at the beginning of the study. The Medical Ethics Committee of the Second Xiangya Hospital, Central South University, approved this study. Raw data in the current research have been reported by previous work of our research team (Yang et al., 2020; Wang et al., 2022; Wu et al., 2022).

Patients were confirmed to meet the DSM-IV (Diagnostic and Statistical Manual of Mental Disorders, 4th edition) criteria for schizophrenia (First and Gibbon, 2004). Exclusion criteria included (1) age less than 16 or greater than 45 years; (2) any contraindications for MRI; (3) history of receiving electroconvulsive therapy; (4) history of alcohol or substance dependence except nicotine; (5) serious physical ailments, organic brain disease (e.g., former stroke, cerebral vascular malformations, and epilepsy), formerly recorded brain injury, chronic neurological illness, or debilitating physical illness; and (6) alcohol use or benzodiazepine treatment, if any, stopped for at least 24 h before scanning.

Clinical symptoms of schizophrenia patients were assessed by the scale for assessment of positive symptoms (SAPS) and the scale for assessment of negative symptoms (SANS) (Phillips et al., 1991). Both patients and HCs were assessed for cognitive functions with the information and digit symbol subscales of the Wechsler Adult Intelligence Scale–Chinese Revised (WAIS-CR) (Gong, 1983) to measure verbal comprehension and processing speed, respectively.

We further divided qualified patients with schizophrenia into three subgroups according to their attention deficit severity, which was measured by the No. 24 item of SANS. The content of the No. 24 item of SANS was “comprehensive severity evaluation of attention” conducted by the interviewer on patients, which mainly included the following aspects: social contact inattention, being absent-minded during the interview, and any other presentation possibly associated with attention deficits (Andreasen, 1989). The score range of this item was 0–5. Thus, we classified the patients based on the score of No. 24 item according to the hierarchy of clinical symptom descriptions (Andreasen, 1989; Phillips et al., 1991). Patients whose No. 24 item score equals 0 or 1 were identified as the mild attention deficit group, patients whose No. 24 item score equals 2 or 3 were identified as the moderate attention deficit group, and patients whose No. 24 item score equals 4 or 5 were identified as the severe attention deficit group.

HCs were recruited and assessed using the Structured Clinical Interview for DSM-IV Axis I Disorders, Research Version, Non-patient Edition (SCID-I/NP). It was confirmed that HCs did not meet any criteria for mental disorders, and their first-degree relatives had no history of any known psychiatric disorders.

### Magnetic resonance imaging data acquisition and preprocessing

The fMRI data were acquired on a Philips Gyroscan Achieva 3.0 T scanner, which had an eight-channel head coil with gradient-recalled echo-planar imaging (EPI) pulse sequence. Participants were asked to perform an N-back WM task during the task-fMRI scanning. Detailed parameter information of the task-fMRI was listed as follows: repetition time (TR) = 2,000 ms, echo time (TE) = 30 ms, flip angle = 90°, field of view (FOV) = 240 × 240 mm^2^, matrix = 64 × 64, slices = 36, slice thickness = 4 mm, gap = 0 mm, and total volumes = 250.

The fMRI data were preprocessed by using the DPABI toolbox (Yan et al., 2016). It consisted of the following steps: discarded several images to reach magnetic saturation, slice timing correction, head motion realignment, spatial normalization to the brain template of Montreal Neurological Institute (MNI) space, smoothing, and linear detrending. Details are seen in Supplementary material 1.

As for the data filtration, the preliminary exclusion criteria of data for preprocessing steps were (1) head motions larger than a 2.5-mm translation or 2.5° rotation in any direction; (2) failure of fMRI data normalization and registration to MNI space due to acquisition errors. Moreover, a number of patients were removed to control the demographic variance (age and gender) among all patient groups. After data quality control, a total of 143 participants, consisting of 92 schizophrenia patients, were grouped by the severity of attention deficits into mild (*n* = 49), moderate (*n* = 29), and severe (*n* = 14), and 51 HCs were enrolled in the subsequent analyses. No statistically significant difference in the total number of displaced volumes for interpolation existed across all groups (*p* = 0.472).

### Working memory task paradigm

**FIGURE 1:**
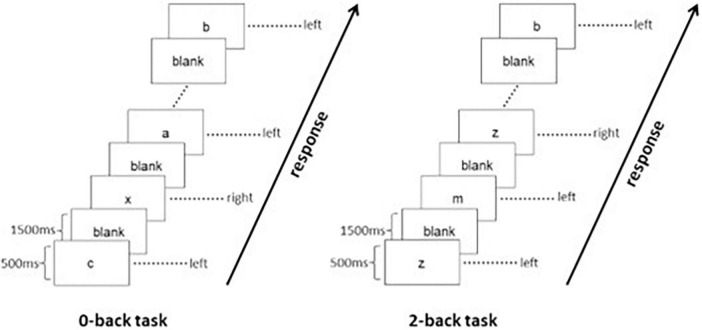
Paradigm of working memory tasks.

The adopted n-back WM task consists of “0-back” and “2-back” loads. Under the “0-back” load, participants were required to press the specific button when they saw the letter “X,” whereas under the “2-back” load, participants were asked to press the specific button when they recognized that the letter shown was identical to the two letters before. The duration of presentation for each letter was 500 ms with an interstimulus interval of 1,500 ms. The stimulation period consisted of “0-back” load and “2-back” load, followed by a resting period during which participants were required to relax and focus on a cross in the screen for 20 s. Stimulation periods and resting periods were shown alternatively. There were four blocks for either “2-back” or “0-back” in this WM task paradigm, and each block included a 2 s guidance and 20 stimulations containing seven targets. The task paradigm is shown in Figure 1. As the “0-back” load is not considered as a qualified WM task, only the volumes during the four blocks of “2-back” load were extracted and concatenated for the construction of the whole-brain functional connectome. We also considered that the BOLD signal has a certain delay, about 5 s (Poldrock et al., 2011), and then shift the extraction window to backward about two volumes when conducting the extracting step. The mean time series was extracted from each of the 264 nodes using 6-mm spheres defined by the Power atlas (Power et al., 2011). A 264 × 264 symmetric matrix was generated for each participant by computing the Pearson correlation coefficients between the time series for each pair of regions of interest (ROIs) and then normalized by the Fisher’s z transformation. We also controlled the variance caused by the effects of age, gender, and education years to derive a corrected matrix.

### Network construction

Network measures at each density (sparsity) were calculated on the Power atlas 264 × 264 weighted adjacency matrices, which were acquired by thresholding the symmetric matrices at a series of network densities, ranging from top 10 to 50% of all connections, with 2% increments, in line with previous studies (Yu et al., 2017; Yang et al., 2020; Tan et al., 2021; Deng et al., 2022). This range density was chosen in the current study for its lower risk of non-biological artifacts and noise (Kaiser and Hilgetag, 2006), and negative correlations were set to zero (Rubinov and Sporns, 2010, 2011). As binarization is arbitrary without widely recognized criteria, which might result in the loss of important illness-related biological features, hence weighted network approaches were applied in our research to avoid this drawback (van den Heuvel et al., 2017; Váša et al., 2018). The Brain Connectivity Toolbox (Rubinov et al., 2009) and the Graph Analysis Toolbox (Hosseini et al., 2012) were applied to quantify network measures and compare the functional networks across all groups, respectively.

### Network properties

Three common network properties of the functional connectome underlying the WM task were subsequently calculated, including the small-worldness, normalized clustering coefficient, and normalized characterized path length. Small-worldness (represented by sigma) was the ratio of normalized clustering coefficient (represented by gamma) to normalized characterized path length (represented by lambda) (i.e., sigma = $\frac{g\,a\,m\,m\,a}{l\,a\,m\,b\,d\,a}$), as normalized topological properties were supposed to be benchmarked against corresponding mean values of null random graphs (i.e., normalized clustering coefficient (gamma) = $\frac{C}{C_{n\,u\,l\,l}}$; normalized characterized path length (lambda) = $\frac{L}{L_{n\,u\,l\,l}}$). Thus, we generated 20 null random networks with the same number of nodes, degree, and degree distribution as the network of interest (Palaniyappan et al., 2015; Das et al., 2018). Based on the network theory, the clustering coefficient (C) of a network was defined as the average of ratio between the actual number of edges between all neighboring nodes and the maximum possible number of edges. The characterized path length (L) of a certain network was defined as the mean value of the shortest path among all pairs of nodes in this network (Achard and Bullmore, 2007; He et al., 2007).

Considering the widely acknowledged hypotheses of function segregation and a growing number of studies focused on relationships between cognition and subnetworks rather than the whole brain (Sheffield et al., 2017; Fransson et al., 2018), we, therefore, further parcellated the whole brain into subnetworks based on the Power atlas to explore the association of WM deficits with subnetworks in schizophrenia with different attention deficits severity. Nodes of each subnetwork were isolated, and topological characteristics of subnetworks were analyzed individually (details of subnetworks are shown in Supplementary material 2).

### Statistical analysis

Group-related differences among demographic, clinical characteristics, and WM task performances were analyzed using a one-way analysis of variance (one-way ANOVA) and chi-square (χ^2^) analysis. *Post hoc* analyses were applied to compare subgroups (patient and HCs groups) alternatively to figure out where difference specifically existed based on the results of the ANOVA test. As network properties were calculated across densities, we first used functional data analysis (FDA) (Bassett et al., 2012) to synthesize values across densities. In the FDA, each network metric curve is treated as a function [y = f (x)], and the sum of differences in y-values is calculated across densities. It is necessary to mention that network metrics calculated on all large-scale networks in our study were generated after the correction of multiple-comparison analysis [false discovery rate (FDR) corrected using the Benjamin–Hochberg method with *p* < 0.05]. Correlation analyses were conducted to further investigate the relationships between the WM task performance, the clinical symptoms severity, and topology properties.

## Results

### Participant characteristics and working memory performance

Demographic, clinical, and WM performance data of all groups are shown in Table 1. Except the education received years (*F* = 11.938, *p* < 0.001), other demographic variables did not differ significantly among all groups. As for the WM task performance, the “2-back” accuracy of the HCs group (84.37 ± 12.63%) was better than all patient groups (*F* = 12.177, *p* < 0.001), and the “2-back” accuracy of schizophrenia patients with mild attention deficits was higher than that of schizophrenia patients with severe attention deficits (*p* = 0.027).

**TABLE 1:** Demographic, clinical, and neurocognitive information.

Variables	Schizophrenia patients (*N* = 92)			HCs (*N* = 51)	F/χ^2^	*P*	*Post hoc* Tukey significance
	Mild (*n* = 49)	Moderate (*n* = 29)	Severe (*n* = 14)				
Age	23.96 ± 5.37	24.14 ± 7.11	23.07 ± 5.23	23.08 ± 4.65	0.356	0.785	N/A
Gender (M/F)	29/20	15/14	11/3	28/23	3.131	0.372	N/A
Education (years)	11.76 ± 2.75	11.40 ± 2.19	12.61 ± 2.69	14.14 ± 1.87	11.938	< 0.001*	HCs > mild: *p* < *0.001; HCs* > *moderate: p* < 0.001
SAPS total score	18.67 ± 12.81	26.62 ± 18.78	30.86 ± 13.82	N/A	4.755	0.011*	Severe > mild: *P* = 0.024
SANS total score	22.06 ± 13.04	58.31 ± 17.09	81.50 ± 12.09	N/A	89.283	< 0.001*	Moderate > mild: *p* < 0.001; severe > mild: *p* < 0.001; severe > moderate: *p* < 0.001
SANS-adapted	20.16 ± 12.53	50.41 ± 16.56	69.50 ± 11.67	N/A	88.825	< 0.001*	Moderate > mild: *p* < 0.001; severe > mild: *p* < 0.001; severe > moderate: *p* < 0.001
Attention deficit score	0.41 ± 0.50	2.76 ± 0.44	4.21 ± 0.43	N/A	462.477	< 0.001*	Moderate > mild: *p* < 0.001; severe > mild: *p* < 0.001; severe > moderate: *p* < 0.001
Total dosage (mg/d)	153.85 ± 512.692	96.56 ± 313.04	1526.73 ± 4275.33	N/A	3.686	0.03*	Severe > mild: *p* < 0.001; severe > moderate: *p* < 0.001
Illness duration (M)	23.59 ± 29.32	23.44 ± 30.08	41.71 ± 40.12	N/A	1.994	0.142	N/A
WAIS-information	17.26 ± 4.81	14.80 ± 4.14	14.25 ± 4.57	N/A	1.513	0.233	N/A
WAIS-Digit symbol	63.24 ± 14.01	61.10 ± 11.93	53.00 ± 9.93	N/A	1.06	0.356	N/A
ACC of 2-back (%)	72.44 ± 14.90	64.69 ± 18.27	61.16 ± 22.41	84.37 ± 12.63	12.177	< 0.001*	HCs > mild: *p* = 0.002; HCs > moderate: *p* < 0.001; HCs > severe: *p* < 0.001; mild > severe: *p* = 0.027
RTC of 2-back (ms)	679.42 ± 127.97	660.21 ± 165.49	592.74 ± 202.87	659.83 ± 134.51	1.148	0.333	N/A

*p < 0.05; n, number; HCs, healthy controls; SAPS, Scale for Assessment of Positive Symptoms; SANS, Scale for Assessment of Negative Symptoms; SANS-adapted, SANS total score without attention items; WAIS_information, information subscale of Wechsler Adult Intelligence Scale-Chinese Revised; WAIS_Digit_Symbol, digit symbol subscale of Wechsler Adult Intelligence Scale-Chinese Revised; N/A, not available; antipsychotic dosage refers to the dose equivalents for chlorpromazine. Bold values indicate statistically significant values.

There are omnibus differences in clinical symptoms among all patient groups [SAPS: *F* = 4.755, *p* = 0.011; SANS-adapted (SANS scale without attention items): *F* = 89.268, *p* < 0.001]. Patients with mild attention deficit have lower SAPS scores than patients with moderate (*p* < 0.001) or severe (*p* = 0.024) attention deficit. As for the negative symptoms except attention deficits, there was a “ladder” pattern of the SANS-adapted among all patient groups (severe > moderate, *p* < 0.001; moderate > mild, *p* < 0.001). Detailed information is presented in Table 1.

### Network properties

**FIGURE 2:**
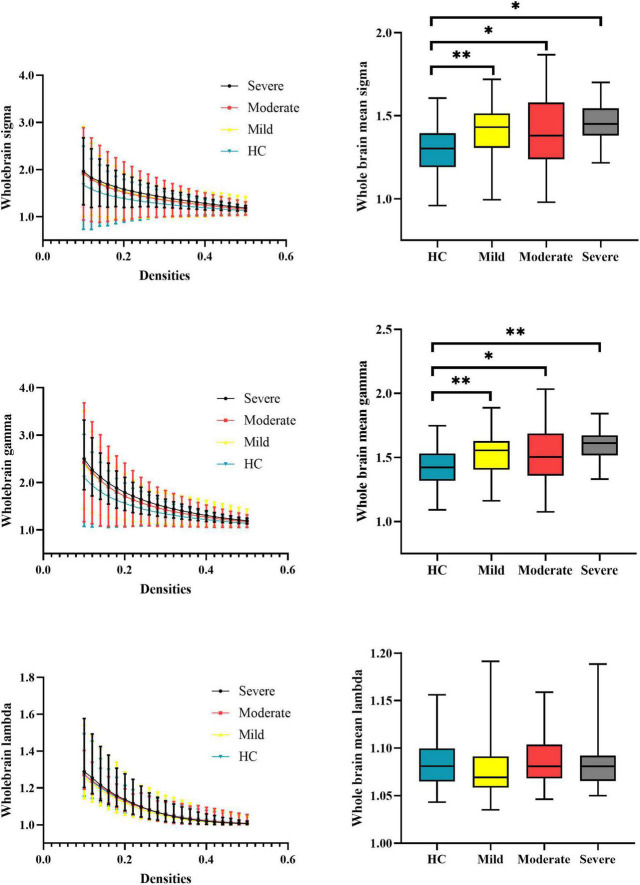
Global properties of the whole-brain functional connectome calculated on the Power atlas of schizophrenia patients with different severities of attention deficits and HCs and comparisons of mean topology properties (sigma, gamma, and lambda) across densities between patients with different severities of attention deficits and HCs. The range of densities is 0.1: 0.02: 0.5, symbol “*” represents *p* < 0.05, and symbol “^**^” represents *p* < 0.01. HCs, healthy controls; sigma, small-worldness; gamma, clustering coefficient; lambda, characterized path length.

At the whole-brain level, one-way ANOVA detected omnibus differences in small-worldness [patients: mild = 1.414 (0.167), moderate = 1.407 (0.210), severe = 1.455 (0.138); HCs = 1.306 (0.148); *p-corrected* = 0.018] and in normalized clustering coefficient [patients: mild = 1.539 (0.173), moderate = 1.541 (0.219), severe = 1.596 (0.132); HCs = 1.426 (0.146); *p-corrected* = 0.014]. The *post hoc* analysis indicated that compared with HCs, patients with mild (small-worldness, *p* = 0.0023; normalized clustering coefficient, *p* = 0.0017), moderate (small-worldness, *p* = 0.0005; normalized clustering coefficient, *p* = 0.0002), and severe (small-worldness, *p* = 0.0154; normalized clustering coefficient, *p* = 0.0061) attention deficit showed aberrantly increased small-worldness and normalized clustering coefficient. Details are presented in Table 2 and Figure 2.

**TABLE 2:** Network properties of functional connectome during WM task.

Measures	Schizophrenia patients			HCs	*F*	*p*	*p*-corrected	*Post hoc* Tukey significance
	Mild	Moderate	Severe					
**Whole-brain**								
Sigma	1.417 (0.167)	1.407 (0.210)	1.455 (0.138)	1.306 (0.148)	5.329	0.002*	0.018*	HCs < mild: *p* = 0.002
								HCs < moderate: *p* < 0.001
								HCs < severe: *p* = 0.015
Gamma	1.539 (0.173)	1.541 (0.219)	1.596 (0.132)	1.426 (0.146)	6.022	0.001*	0.011*	HCs < mild: *p* = 0.002
								HCs < moderate: *p* < 0.001
								HCs < severe: *p* = 0.006
Lambda	1.077 (0.030)	1.086 (0.025)	1.088 (0.036)	1.086 (0.026)	1.125	0.341	0.614	N/A
**VAN**								
Sigma	1.494 (1.301)	1.455 (0.788)	1.788 (1.329)	1.389 (0.638)	0.580	0.629	0.830	N/A
Gamma	1.304 (0.599)	1.325 (0.482)	1.408 (0.600)	1.264 (0.389)	0.316	0.814	0.907	N/A
Lambda	0.985 (0.067)	0.967 (0.073)	0.933 (0.130)	0.972 (0.074)	1.661	0.178	0.486	N/A
**SBN**								
Sigma	1.815 (0.976)	1.858 (0.785)	1.884 (0.761)	Null	0.044	0.957	0.957	N/A
Gamma	1.631 (0.482)	1.666 (0.482)	1.750 (0.469)	Null	0.339	0.714	0.838	N/A
Lambda	0.948 (0.073)	0.949 (0.086)	0.977 (0.054)	0.978 (0.062)	2.064	0.108	0.486	N/A
**SSHN**								
Sigma	1.489 (0.232)	1.515 (0.295)	1.579 (0.198)	1.468 (0.257)	0.774	0.511	0.812	N/A
Gamma	1.642 (0.288)	1.659 (0.290)	1.793 (0.276)	1.616 (0.324)	1.284	0.282	0.614	N/A
Lambda	1.103 (0.047)	1.121 (0.051)	1.127 (0.036)	1.109 (0.051)	1.455	0.230	0.565	N/A
**SN**								
Sigma	1.612 (0.739)	1.437 (0.428)	1.638 (0.511)	1.447 (0.377)	1.190	0.316	0.614	N/A
Gamma	1.523 (0.389)	1.426 (0.360)	1.626 (0.359)	1.423 (0.309)	1.701	0.170	0.486	N/A
Lambda	1.029 (0.076)	1.031 (0.060)	1.052 (0.031)	1.025 (0.057)	0.656	0.581	0.830	N/A
**FPN**								
Sigma	1.314 (0.282)	1.356 (0.301)	1.392 (0.269)	1.408 (0.216)	1.141	0.335	0.614	N/A
Gamma	1.378 (0.294)	1.444 (0.318)	1.530 (0.354)	1.501 (0.242)	1.914	0.130	0.486	N/A
Lambda	1.066 (0.054)	1.078 (0.052)	1.100 (0.062)	1.077 (0.045)	1.653	0.180	0.486	N/A
**DAN**								
Sigma	1.439 (0.541)	1.322 (0.366)	1.374 (0.457)	1.341 (0.367)	0.579	0.630	0.830	N/A
Gamma	1.344 (0.361)	1.274 (0.270)	1.290 (0.319)	1.268 (0.294)	0.552	0.647	0.830	N/A
Lambda	0.964 (0.082)	0.967 (0.061)	0.969 (0.062)	0.974 (0.075)	0.149	0.930	0.957	N/A
**DMN**								
Sigma	1.266 (0.172)	1.321 (0.186)	1.392 (0.152)	1.321 (0.193)	2.026	0.113	0.486	N/A
Gamma	1.411 (0.196)	1.481 (0.213)	1.607 (0.186)	1.470 (0.221)	3.371	0.020*	0.135	N/A
Lambda	1.108 (0.029)	1.114 (0.035)	1.143 (0.049)	1.104 (0.026)	5.863	0.001*	0.011*	HCs < severe: *p* < 0.001
								Mild < severe: *p* = 0.002
								Moderate < severe: *p* = 0.029
**CON**								
Sigma	1.558 (0.488)	1.621 (0.481)	1.473 (0.343)	1.719 (0.855)	0.815	0.488	0.812	N/A
Gamma	1.480 (0.323)	1.523 (0.372)	1.441 (0.310)	1.577 (0.631)	0.510	0.676	0.830	N/A
Lambda	0.988 (0.077)	0.985 (0.065)	0.993 (0.057)	0.978 (0.055)	0.279	0.840	0.907	N/A

*p < 0.05. VAN, ventral attention network; SBN, subcortical network; SSHN, sensory somatomotor hand network; SN, salience network; FPN, frontoparietal network; DAN, dorsal attention network; DMN, default-mode network; CON, cingulo-opercular network; sigma, small-worldness; gamma, clustering coefficient; lambda, characterized path length. Bold values indicate statistically significant values.

**FIGURE 3:**
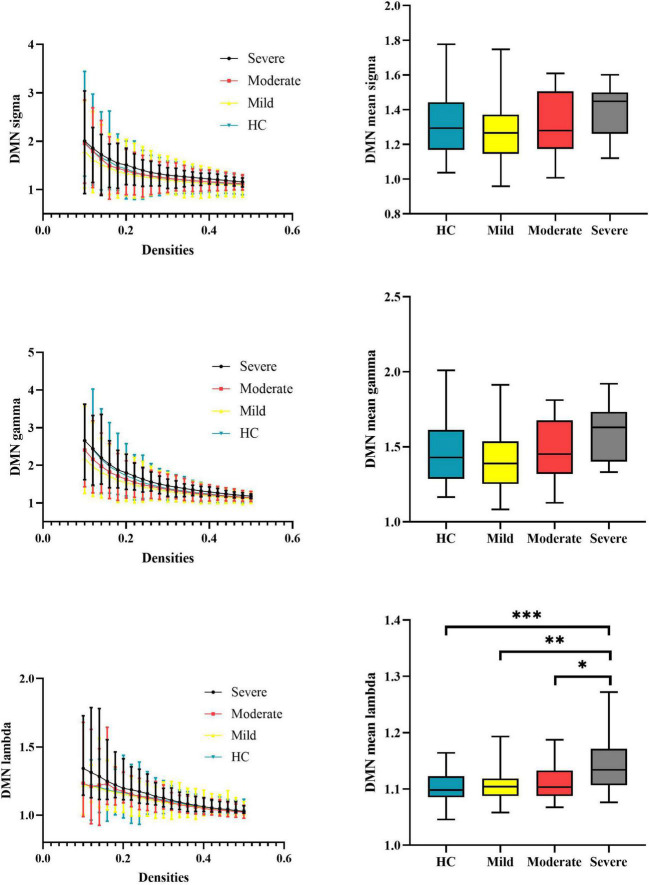
Global properties of the DMN functional connectome calculated on Power atlas of schizophrenia patients and HCs and comparisons of mean topology properties (sigma, gamma, and lambda) across densities between schizophrenia patients with different severities of attention deficits and HCs. The range of densities is 0.1: 0.02: 0.5, symbol “*” represents *p* < 0.05, and symbol “^**^” represents *p* < 0.01. DMN, default-mode network; HCs, healthy controls; sigma, small-worldness; gamma, clustering coefficient; lambda, characterized path length. ***P < 0.001.

At the subnetwork level, one-way ANOVA detected omnibus differences in normalized characterized path length of the DMN [patients, mild = 1.108 (0.029), moderate = 1.114 (0.035), severe = 1.143 (0.049); HCs = 1.104 (0.026); *p-corrected* = 0.014]. The *post hoc* analysis indicated that patients with severe attention deficits showed increased normalized characterized path length of the DMN compared with HCs (*p* < 0.001), patients with mild attention deficits (*p* = 0.0021), and patients with moderate attention deficits (*p* = 0.029). Details are presented in Table 2 and Figure 3.

We further explored whether the detected network properties of the whole-brain functional connectome and DMN subnetwork showed omnibus across all groups during the resting state. Detailed scanning parameters, data preprocessing, and network construction information of resting fMRI are shown in Supplementary material 3. However, we did not observe any omnibus differences in the network properties of the whole-brain functional connectome and DMN subnetwork under the resting state (see Table 3).

**TABLE 3:** Network properties of functional connectome during resting state.

Measures	Schizophrenia patients			HCs	*F*	*p*
	Mild	Moderate	Severe			
**Whole-brain**						
Sigma	1.468 (0.206)	1.460 (0.182)	1.420 (0.129)	1.415 (0.242)	0.529	0.663
Gamma	1.584 (0.213)	1.585 (0.184)	1.550 (0.118)	1.540 (0.242)	0.397	0.756
Lambda	1.072 (0.035)	1.076 (0.027)	1.083 (0.048)	1.083 (0.045)	0.728	0.538
**DMN**						
Sigma	1.265 (0.162)	1.312 (0.171)	1.371 (0.136)	1.305 (0.173)	1.157	0.329
Gamma	1.408 (0.193)	1.477 (0.224)	1.528 (0.127)	1.455 (0.203)	1.151	0.331
Lambda	1.106 (0.028)	1.117 (0.029)	1.115 (0.064)	1.098 (0.024)	2.157	0.097

DMN, default-mode network; sigma, small-worldness; gamma, clustering coefficient; lambda, characterized path length.

### Correlation analyses

**FIGURE 4:**
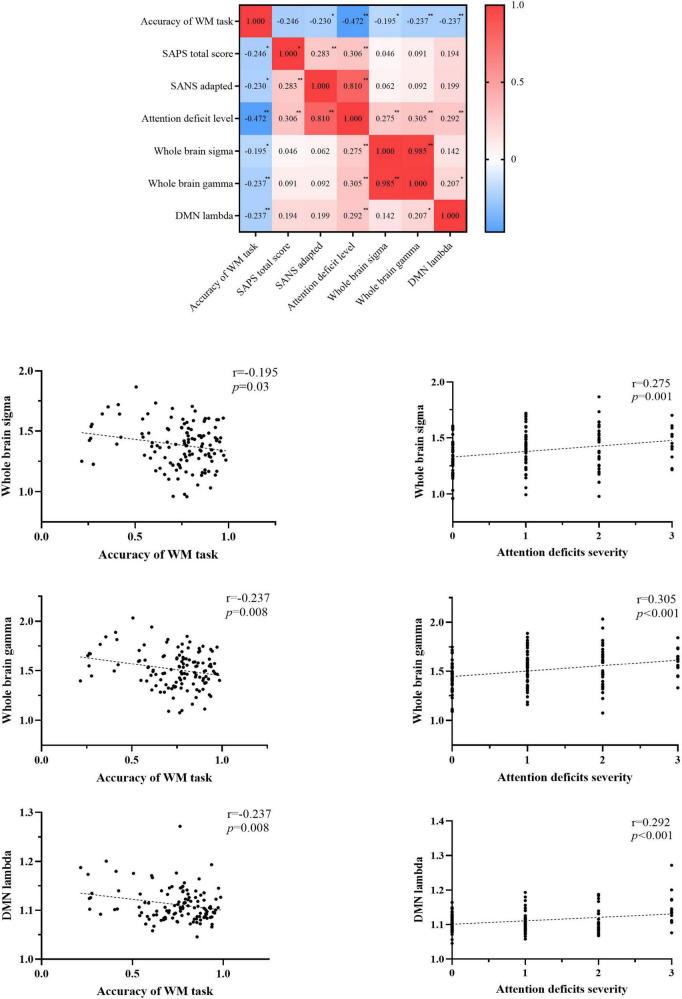
Correlations among the accuracy of the WM task, clinical symptoms, and topology properties. WM, working memory; SAPS, scale for assessing positive symptoms; SANS, scale for assessment of negative symptoms; SANS-adapted, SANS total score without attention items; DMN, default-mode network; sigma, small-worldness; gamma, clustering coefficient; lambda, characterized path length. *P < 0.05, **P < 0.01, ***P < 0.001.

We observed the decreased accuracy of the WM task was correlated with increased clinical symptoms (SANS-adapted, *r* = –0.230, *p* = 0.041; SAPS, *r* = –0.246, *p* = 0.029; attention deficit level, *r* = –0.472, *p* < 0.001), increased topology properties (small-worldness, *r* = –0.195, *p* = 0.03; normalized clustering coefficient, *r* = –0.237, *p* = 0.008) of the whole brain, and increased normalized characterized path length of the DMN network (*r* = –0.237, *p* = 0.008). The topology properties significantly related to the attention deficit level were whole-brain small-worldness (*r* = 0.275, *p* = 0.001), whole-brain normalized clustering coefficient (*r* = 0.305, *p* < 0.001), and DMN normalized characterized path length (*r* = 0.292, *p* < 0.001). Details are presented in Figure 4 and Supplementary material 4.

## Discussion

To our best knowledge, this is the first study to investigate the topology of the functional connectome during the WM task in schizophrenia with different attention deficit severity. We reported three key findings. First, all patient groups showed increased small-worldness and local clustering of the whole-brain functional connectome compared with HCs, despite their varied attention deficit severity. Second, patients with severe attention deficits showed decreased global integration of the DMN network compared with HCs and patients with less severe attention deficits. Third, the abovementioned findings in patients with schizophrenia under the WM task were not manifested under the resting state.

In line with our prior work (Yang et al., 2020), this study reported that all patients with different attention deficit severity showed increased small-worldness and normalized clustering coefficient of their whole-brain functional connectome during the WM task. In patient groups, the increased small-worldness is driven by higher local clustering but not the global integration. This topology alteration was similar to the ketamine-induced alteration of the functional connectome (Dawson et al., 2014), which was commonly seen among the pharmacological models of the behavioral deficits similar to schizophrenia in both humans (Morgan et al., 2004; D’Souza et al., 2012) and animals (Roberts et al., 2010; Skoblenick and Everling, 2012). The local clustering is essential for motor execution, whereas the global integration is vitally important for WM (Sakreida et al., 2018; Bhattacharjee and Schwarz, 2022). It is an inefficient way for patients with schizophrenia to promote their WM performance by improving their local clustering (Yang et al., 2020). Furthermore, we did not observe any differences in the topology properties of the whole-brain functional connectome among all patient groups. This finding may collaborate with our prior study and demonstrate that the increased local clustering under the WM task is more likely a “trait” marker of patients with schizophrenia, but irrelevant to their clinical symptoms.

Patients with severe attention deficits showed an increased normalized characterized path length of the DMN network compared with HCs and patients with mild attention deficits. Our results suggested that the efficiency of DMN might be positively meaningful when the demands on WM capacity increased (Spreng, 2012; Sormaz et al., 2018; Buckner and DiNicola, 2019). From the perspective of topology structure, the increase in normalized characterized path length indicated the worse global efficiency of DMN. Patients with severe attention deficits need to devote more neural resources to achieve the global transition of information in DMN. The increase in normalized characterized path length in DMN was correlated with the increase in the attention deficit degree among patients. This result could be attributed to the patients with severe attention deficits cannot timely respond to support the flexible topology reconfiguration when facing the demand of WM task (Moraschi et al., 2020). Thus, we speculated that the inefficiency of DMN among patients with severe attention deficits affects the processing of WM in these certain phases and then causes worse WM task performance than that of HCs.

The decreased integration of the DMN was only observed in schizophrenia with severe attention deficits, which may demonstrate that the DMN can be a potential target for clinical intervention. This speculation was also supported by previous research on attention-deficit/hyperactivity disorder (ADHD), a mental disorder that mainly includes attention deficits and an overload of cognitive process (Klingberg, 2009). Bachmann et al. (2018) have demonstrated that short-term mindfulness could significantly improve WM task performance in ADHD patients. Mindfulness is a widely approved psychological intervention for ADHD. It has been considered closely associated with DMN, and DMN has been recognized as a potential biomarker for monitoring the treatment effects of mindfulness (Simon and Engström, 2015).

It should be noted that there were no omnibus significant brain connectome topology alterations among all groups under resting state. Indeed, previous studies have reported significant differences of brain network topology between patients with schizophrenia and HCs. For example, Yu et al. (2017) and Jiang et al. (2022) have reported a lower clustering coefficient and small-worldness in schizophrenia compared with HCs. However, some studies reported inconsistent findings. For instance, Hadley et al. (2016) have reported the increased clustering coefficient but deceased global efficiency in the unmediated schizophrenias during resting state compared with HCs, and this pattern of aberrant functional network integration and segregation in responders can be modulated with 6-week risperidone treatment. We speculated this inconsistency may be due to the heterogeneity of the enrolled participants. However, it should be noted that the omnibus differences in network properties across all groups were detected during the WM task. It may suggest that the alteration of network properties was context-dependent. During the resting state, participants were only asked to keep their eyes open and not fall asleep. In contrast, during the WM task, attention resources need to be sufficiently devoted to accomplishing this task. Concerned about the dynamic switch of topological configurations of the DMN observed during the WM task (Duan et al., 2019; Moraschi et al., 2020), we speculated that the process of attention mobilization acted as an external inducement for the functional connectome topology alteration. This finding may be consistent with previous studies conducted in HCs, which indicated that the dynamic topology reorganization of DMN is associated with the WM task (Tommasin et al., 2018; Moraschi et al., 2020).

## Limitation

There are some limitations in the current study. First, the proportion of schizophrenia patients with different severity of attention deficits is varied, especially the limited sample size of schizophrenia patients with severe attention deficits. We, therefore, suggest that these preliminary results of the current study need to be validated in a future big dataset. Second, the discrepancy in SANS total score among the schizophrenia patients with severe attention deficits and the other two patient groups was apparent. Concerned that the criterion of attention deficits applied in our research was a part of the SANS scale, which was associated with other negative symptoms like avolition or blunted effect, we considered that it was reasonable that other negative symptoms decrease the efficiency of attention allocation. So, it is hard to exclude the effect of the negative symptoms on the normalized characterized path length of DMN. Thus, we conducted the correlation analyses among SANS-adapted (SANS total score without attention items) and topology properties, which were related to the attention deficits. However, there were no significant relationships among these variables. Third, the adopted N-back paradigm lacked various WM loads, and we were not able to further explore the differences in functional connectome topology of schizophrenia patients with diverse attention deficits and HCs under varied WM loads.

## Conclusion

This is the first study to investigate the topology of the functional connectome during a WM task in schizophrenia with different attention deficit severity. All patients with schizophrenia showed higher small-worldness that was induced by increased clustering compared with HCs. The decreased integration of DMN is associated with severe attention deficits in patients with schizophrenia, while these topology alterations were absent in schizophrenia patients with severe attention deficits during resting state. The current study suggested that the mobilization of attention resources might be an external inducement of the topology reorganization of the DMN during the N-back WM task among schizophrenia patients with severe attention deficits.

## Data availability statement

The raw data supporting the conclusions of this article will be made available by the authors, without undue reservation.

## Ethics statement

The studies involving human participants were reviewed and approved by the Medical Ethics Committee of the Second Xiangya Hospital, Central South University. Written informed consent to participate in this study was provided by the participants’ legal guardian/next of kin.

## Author contributions

ZL and JiY designed the study. JuY, FS, ZF, and PC acquired the data. PC, JiY, and ZF analyzed the data. PC and JiY wrote the manuscript. All authors contributed to the article and approved the submitted version.
