# Supplementary material for: Decreased integration of default-mode network during a working memory task in schizophrenia with severe attention deficits
Source: Front Cell Neurosci. 2022 Nov 8;16:1006797. doi: 10.3389/fncel.2022.1006797 (PMC9679280; doi:10.3389/fncel.2022.1006797)
Supplement: Supplementary file 1 [file Data_Sheet_1.pdf]

Supplementary materials

S1. Detailed information of task fMRI data preprocessing

S2. Detailed information in calculating network properties of subnetworks

S3. Detailed analysis information of resting-state fMRI

S4. Correlation analyses of accuracy of WM task, clinical symptoms, and topology properties

S5. References for Supplementary materials

S1. Detailed information of fMRI data preprocessing

Firstly, we removed the first 2 volumes to avoid insufficient magnetic saturation, and 248 volumes of each participant were kept for entering the remaining preprocessing steps. The remaining preprocessing steps included slice timing correction, head motion realignment, smoothing (full width at half maximum [FWHM]=8mm), and spatial normalization to the brain template of Montreal Neurological Institute (MNI) space. Nuisance covariates, including 12 head motion parameters, white matter, and ventricle signals were regressed out. Considering that illness-related variance might exist in the global signals, thus global signals were retained in the analysis. Nearest-neighbor interpolation was used for the displaced volumes (framewise displacement>0.5 mm). The exclusion criteria of data for preprocessing steps were (1) head motions larger than a 2.5-mm translation or 2.5° rotation in any direction; (2) Failure of fMRI data normalization and registration to MNI space due to acquisition errors. There were no statistically significant differences in total number of displaced volumes that underwent interpolation in patients and controls.

S2. Detailed information in calculating network properties of subnetworks

The 264 nodes of the whole-brain defined by the Power atlas can be parcellated into 14 subnetworks including 'auditory', 'visual', 'cingulo-opercular', 'default mode', 'dorsal attention', 'frontoparietal', 'salience network', 'sensory/somatomotor hand', 'subcortical network', 'ventral attention', 'sensory/somatomotor mouth', 'cerebellar', 'uncertain', and 'memory retrieval' network.

We isolated the nodes of each subnetwork and separately calculated their sigma, gamma, and lambda on a series of weighted adjacency matrices with different densities, ranging from top 10% to 50% of all connections, with 2% increments. 4 subnetworks, 'sensory/somatomotor mouth', 'cerebellar', 'uncertain', and 'memory retrieval' network, had insufficient number of nodes to meaningfully calculate sub-graph proporties and thus were excluded from further analysis. The detailed steps of the following statistical analysis are in line with the statistical analysis described in our main document.

S3. Detailed analysis information of resting-state fMRI

Scanning parameters: Resting state fMRI data was obtained by a Philips Gyroscan Achieva 3.0 T scanner using a gradient-recalled echo-planar imaging pulse sequence. During the scanning process, participants were instructed to maintain a 'resting state' neutral position, including having a flat, still body, remaining awake, opening eyes, and do not think about any particular idea. The specific scanning sequence and parameters are as follows: (36 slice, matrix = 64 × 64, repetition time (RT) = 2000 ms, flip angle (FA) = 90°, echo time (TE) = 30 ms, gap = 0 mm, slice thickness = 4 mm, 250 total volumes).

Data preprocessing: We removed the first 10 volumes to avoid insufficient magnetic saturation, and 240 volumes of each participant were kept for entering the remaining preprocessing steps. The remaining preprocessing steps included slice timing correction, head motion realignment, smoothing (full width at half maximum [FWHM]=8mm), and spatial normalization to the brain template of Montreal Neurological Institute (MNI) space. Nuisance covariates, including 12 head motion parameters, white matter, and ventricle signals were regressed out. Considering that illness-related variance might exist in the global signals (1), thus global signals were retained in the analysis. Nearest-neighbor interpolation was used for the displaced volumes (framewise displacement>0.5 mm). The exclusion criteria of data for preprocessing steps were (1) head motions larger than a 2.5-mm translation or 2.5° rotation in any direction; (2) Failure of fMRI data normalization and registration to MNI space due to acquisition errors.

Network construction: The mean time series was extracted from each of the 264 nodes using 6-mm spheres defined by the Power atlas (2). A 264 × 264 symmetric matrix was generated for each participant by computing the Pearson correlation coefficients between the time series for each pair of regions of interest (ROIs), and then normalized by the Fisher's z transformation. We also controlled the variance caused by the effects of age, gender, and education years to derive corrected matrix.

Identical with previous studies, network properties at each density (sparsity) were calculated on the Power atlas 264 × 264 weighted adjacency matrices, which were

acquired by thresholding the symmetric matrices at a series of network densities, ranging from top 10% to 50% of all connections, with 2% increments, which were chosen for its lower risk of nonbiological artifacts and noise. Negative correlations were set to zero (3, 4). Weighted network approaches were applied in the current research to avoid loss of important illness-related biological features. The Brain Connectivity Toolbox (5) and the Graph Analysis Toolbox (6) were applied to quantify network measures and compare the functional networks across all groups, respectively.

S4. Correlation analyses of accuracy of WM task, clinical symptoms, and topology properties

	Accuracy of WM task	SAPS total score	SANS adapted	Attention deficit level	Whole brain sigma	Whole brain gamma	DMN lambda
Accuracy of WM task	1	r=-0.246*<br>*P*=0.029	r=-0.230*<br>*P*=0.041	r=-0.472**<br>*P*<0.001	r=-0.195*<br>*P*=0.030	r=-0.237**<br>*P*=0.008	r=-0.237**<br>*P*=0.008
SAPS total score	r=-0.246*<br>*P*=0.029	1	r=0.283**<br>*P*=0.006	r=0.306**<br>*P*=0.003	r=0.046<br>*P*=0.046	r=0.091<br>*P*=0.391	r=0.194<br>*P*=0.064
SANS adapted	r=-0.230*<br>*P*=0.041	r=0.283**<br>*P*=0.006	1	r=0.810**<br>*P*<0.001	r=0.062<br>*P*=0.559	r=0.092<br>*P*=0.382	r=0.199<br>*P*=0.058
Attention deficit severity	r=-0.472**<br>*P*<0.001	r=0.306**<br>*P*=0.003	r=0.810**<br>*P*<0.001	1	r=0.275**<br>*P*=0.001	r=0.305**<br>*P*<0.001	r=0.292**<br>*P*<0.001
Whole brain sigma	r=-0.195*<br>*P*=0.03	r=0.046<br>*P*=0.664	r=0.062<br>*P*=0.559	r=0.275**<br>*P*=0.001	1	r=0.985**<br>*P*<0.001	r=0.142<br>*P*=0.091
Whole brain gamma	r=-0.237**<br>*P*=0.008	r=0.091<br>*P*=0.391	r=0.092<br>*P*=0.382	r=0.305**<br>*P*<0.001	r=0.985**<br>*P*<0.001	1	r=0.207*<br>*P*=0.013
DMN lambda	r=-0.237**<br>*P*=0.008	r=0.194<br>*P*=0.064	r=0.199<br>*P*=0.058	r=0.292**<br>*P*<0.001	r=0.142<br>*P*=0.091	r=0.207*<br>*P*=0.013	1

Note: WM: working memory; SAPS: scale for assessing positive symptoms; SANS: scale for assessment of negative symptoms; SANS adapted: SANS total score without attention items; DMN: default mode network.

S5. References for Supplementary materials:

1. Yang GJ, Murray JD, Repovs G, Cole MW, Savic A, Glasser MF, et al. Altered global brain signal in schizophrenia. Proceedings of the National Academy of Sciences of the United States of America. 2014;111(20):7438-43.doi: 10.1073/pnas.1405289111
2. Power JD, Cohen AL, Nelson SM, Wig GS, Barnes KA, Church JA, et al. Functional network organization of the human brain. Neuron. 2011;72(4):665-78.doi: 10.1016/j.neuron.2011.09.006
3. Rubinov M, Sporns O. Complex network measures of brain connectivity: uses and interpretations. NeuroImage. 2010;52(3):1059-69.doi: 10.1016/j.neuroimage.2009.10.003
4. Rubinov M, Sporns O. Weight-conserving characterization of complex functional brain networks. NeuroImage. 2011;56(4):2068-79.doi: 10.1016/j.neuroimage.2011.03.069
5. Rubinov M, Kötter R, Hagmann P, Sporns O. Brain connectivity toolbox: a collection of complex network measurements and brain connectivity datasets. NeuroImage. 2009(47):S169.doi,
6. Hosseini SH, Hoeft F, Kesler SR. GAT: a graph-theoretical analysis toolbox for analyzing between-group differences in large-scale structural and functional brain networks. PloS one. 2012;7(7):e40709.doi,
